# Single-Cell Analysis of Molecular Mechanisms in Rapid Antler Osteogenesis During Growth and Ossification Stages

**DOI:** 10.3390/ijms26062642

**Published:** 2025-03-14

**Authors:** Ranran Zhang, Xiumei Xing

**Affiliations:** 1 Institute of Special Animal and Plant Sciences, Chinese Academy of Agricultural Sciences, Changchun 130112, China; heavenranran@163.com; 2 Key Laboratory of Genetics, Breeding and Reproduction of Special Economic Animal, Ministry of Agriculture and Rural Affairs of the People’s Republic of China, Changchun 130112, China

**Keywords:** antler, scRNA-seq, bulk RNA-seq, osteogenesis

## Abstract

Antlers, as the only fully regenerable bone tissue in mammals, serve as an exceptional model for investigating bone growth, mineralization, articular cartilage repair, and the pathophysiology of osteoporosis. Nevertheless, the exact molecular mechanisms governing osteogenesis, particularly the dynamic cellular interactions and signaling pathways coordinating these processes, remain poorly characterized. This study used single-cell RNA sequencing (scRNA-seq) on the 10× Genomics Chromium platform, combined with bulk-RNA sequencing results, to comprehensively analyze molecular regulatory mechanisms in rapid antler osteogenesis. The results showed that eight cell types were identified in sika deer antler during the growth and ossification stages: mesenchymal, chondrocyte, osteoblast, pericyte, endothelial, monocyte/macrophage, osteoclast, and NK cells. Chondrocytes were predominantly found during the growth stage, while osteoblasts were more abundant during the ossification stage. Mesenchymal cells were subclassified into three subcategories: MSC_1 (*VCAN* and *SFRP2*), MSC_2 (*TOP2A*, *MKI67*), and MSC_3 (*LYVE1* and *TNN*). MSC_3 was predominantly present during the growth stage. During the growth stage, MSC_1 and MSC_2 upregulated genes related to vasculature development (*COL8A1*, *NRP1*) and cell differentiation (*PTN*, *SFRP2*). During the ossification stage, these subcategories upregulated genes involved in the positive regulation of p53 class mediator signal transduction (*RPL37*, *RPL23*, *RPS20*, and *RPL26*), osteoblast differentiation (*SPP1*, *IBSP*, *BGLAP*), and proton-motive ATP synthesis (*NDUFA7*, *NDUFB3*, *NDUFA3*, *NDUFB1*). Endothelial cells were categorized into five subpopulations: Enc_1 (*SPARCL1*, *VWF*), Enc_2 (*MCM5*), Enc_3 (*ASPM*, *MKI67*), Enc_4 (*SAT1*, *CXCL12*), and Enc_5 (*ZFHX4*, *COL6A3*). Combined scRNA-seq and bulk RNA-seq analysis revealed that the ossification stage’s upregulation genes included osteoclast- and endothelial cell-specific genes, while the growth stage’s upregulation genes were mainly linked to collagen organization, osteoblast differentiation, mitotic cell cycle, and chondrocyte differentiation. Overall, this study offers a detailed single-cell analysis of gene expression patterns in antlers during the growth and ossification stages, providing insights into the molecular mechanisms driving rapid osteogenesis.

## 1. Introduction

The antler, a unique appendage on male deer, represents a fully regenerative bone tissue in mammals. Its extraordinary growth rate makes it an invaluable model for investigating bone growth, cartilage repair, and osteoporosis pathophysiology [1]. Antler regeneration involves two primary osteogenic patterns: early intramembranous ossification responsible for diameter expansion, and endochondral ossification driving longitudinal growth. The process can be divided into two distinct stages. The first stage (growth stage) begins with the continuous proliferation of mesenchymal stem cells at the distal end of the antlers. These cells progressively differentiate into chondroblasts, chondrocytes, and hypertrophic chondrocytes, enabling sustained cartilage formation. The second stage (rapid ossification stage) is marked by gradual hardening of the initially soft antler, a process which has been hypothesized to be triggered by elevated testosterone levels [2].

Antler formation relies on stem cells residing in the antlerogenic periosteum. These cells not only express mesenchymal stem cell markers (*CD29*, *CD90*, *NPM1*, *STRO-1*) but also exhibit markers characteristic of embryonic stem cells (*CD9*, *VIM*) [3,4]. Additionally, these progenitor cells demonstrate elevated expression of genes associated with angiogenesis, neurogenesis, and intramembranous ossification [5,6]. In vitro studies confirm that these cells possess pluripotent stem cell properties, with the capacity to differentiate into fibroblasts, osteoblasts, chondrocytes, adipocytes, and skeletal muscle cells [3,7]. The diverse differentiation directions of mesenchymal stem cells are regulated by distinct transcription factors. *SOX9*, a marker of antler chondrocyte progenitor cells, plays a critical role in the sequential events of cartilage formation by regulating a cascade of downstream factors [8,9,10]. *Runx2*, predominantly expressed in preosteoblasts and immature osteoblasts, directs the differentiation of pluripotent stem cells into osteoblasts by directly regulating downstream factors such as *sp7* and pathways such as Hedgehog, Fgf, and Wnt [11,12]. Additionally, *Runx2* inhibits apoptosis in terminal hypertrophic chondrocytes and induces their transdifferentiation into osteoblast lineage cells [13].

With the advancement of sequencing technologies, various omics approaches have been increasingly applied to elucidate the biological mechanisms underlying antler development. Wang employed genomic and transcriptomic approaches, revealing a significant correlation in the gene expression profiles between antler tissue and human osteosarcoma tissue [14]. Liu conducted metabolomic analyses, discovering that uridine can significantly reverse human stem cell aging and promote the regeneration of multiple tissues [15]. Through scRNA-seq analysis, Qin identified antler blastema progenitor cells (ABPCs) originating from PRRX1+ mesenchymal cells, which play a pivotal role in driving antler regeneration [16]. However, research on the rapid osteogenesis mechanisms of deer antlers remains relatively limited. Our previous transcriptomic and proteomic analyses revealed that chondrocyte development genes were upregulated during the growth stage, while genes related to osteoblast differentiation, retinol metabolism, and immune response were downregulated during the ossification stage [17,18]. This observation piqued our interest: during the transition from the rapid growth stage to the ossification stage, how do alterations in cell proliferation and differentiation contribute to the cessation of antler growth and accelerated ossification? Are these changes correlated with fluctuations in hormone levels, particularly testosterone?

In this study, we collected antler tissue at 60 days (growth stage) and 90 days (ossification stage) and used the 10× Genomics single-cell platform to map the single-cell transcriptome atlas of the growth center cells. By comparing cell composition and gene expression differences between these stages, we explored the differentiation of mesenchymal cells, chondrocytes, and osteoblasts and integrated previous transcriptome data to identify key factors regulating rapid ossification. This study aims to reveal the molecular mechanisms of rapid antler osteogenesis and provide theoretical insights for mammalian bone growth and ossification research.

## 2. Results

### 2.1. Antler Single-Cell RNA Sequencing

Single-cell suspensions from two antler samples were prepared and evaluated. The viabilities were 84% and 93%, and the concentrations were 965 cells/μL and 889 cells/μL, meeting the quality criteria. The 10× Genomics Chromium platform was then used for single-cell capture, labeling, library construction, and sequencing. The results showed that 13,941 cells from the growth stage had an average of 76,925 reads per cell, with 94.3% alignment to the sika deer reference genome. For the ossification stage, 8793 cells averaged 125,636 reads per cell, with 94.8% alignment. After further removing low-quality cells using Seurat, 12,512 and 8496 cells were retained from the growth and ossification stages, respectively.

### 2.2. Cell Annotation

**Figure 1:**
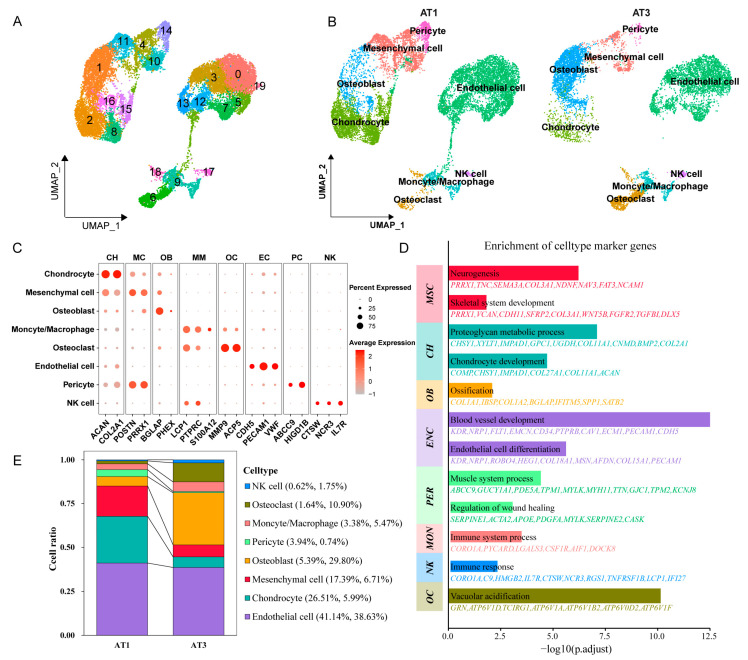
Single-cell transcriptome sequencing of sika deer antler during the growth and ossification stages. (**A**): The UMAP plot of the scRNA-seq analysis of antlers, with different clusters labeled by distinct colors. (**B**): Cell type annotation results based on marker genes, indicating the distribution of various cell types within the UMAP plot. (**C**): Bubble plot showcasing the marker genes for individual cell types. The size of the bubbles represents the percentage of cells expressing the gene, while the color intensity indicates the average expression level. (**D**): Functional enrichment analysis of the marker genes (|avg_log2FC| > 2 and *p*_values < 0.05) for each cell type. (**E**): Bar chart depicting the percentage of each cell type across different samples.

Seurat software performed data standardization, followed by dimensionality reduction and clustering to identify 20 cell clusters (Figure 1A, Table S1). To characterize the biological features of these clusters, we screened for highly variable genes in each cluster and identified specific marker genes using the Cellmarker 2.0 database and the relevant literature. This process allowed us to distinguish eight cell types: mesenchymal cell (*POSTN*, *PRRX1*), chondrocyte (*ACAN*, *COL2A1*), osteoblast (*BGLAP*, *PHEX*), pericyte (*ABCC9*, *HIGD1B*), endothelial cell (*PECAM1*, *VWF*), monocytes/macrophage (*LCP1*, *S100A12*), osteoclast (*MMP9*, *ACP5*), and NK cell (*CTSW*, *NCR3*) (Figure 1B,C, Table S2). Notably, cluster19 containing only 43 cells could not be confidently classified into any of these cell types and was excluded from subsequent analyses.

Furthermore, we conducted functional enrichment analysis on the marker genes (|avg_log2FC| > 2 and *p*_values < 0.05) for each cell type, revealing cell type-specific associations with diverse biological processes (Figure 1D). The marker genes of mesenchymal cells were significantly enriched in neurogenesis (*p =* 6.09 × 10^−7^) and skeletal system development (*p =* 0.0152), suggesting their critical role in neural development during the antler growth stage. Chondrocytes exhibited enrichment in cartilage development (*p =* 1.94 × 10^−5^), while osteoblasts were linked to ossification (*p =* 0.0081). Endothelial cell markers were significantly associated with endothelial cell differentiation (*p =* 2.39 × 10^−6^) and blood vessel development (*p =* 3.16 × 10^−13^). Furthermore, pericyte markers showed a strong association with the regulation of wound healing (*p =* 0.00078), indicating synergistic interactions between pericytes and endothelial cells in promoting vascular formation during antler regeneration. Osteoclast markers were associated with vacuolar acidification (*p =* 7.24 × 10^−11^), a process essential for their bone resorption function.

Significant differences in cell distribution were observed between AT1 and AT3 samples (Figure 1E). In AT1, mesenchymal cells and chondrocytes accounted for 17.39% and 26.51%, respectively, decreasing to 6.71% and 5.99% in AT3. Conversely, osteoblasts and osteoclasts increased from 5.39% and 1.64% in AT1 to 29.80% and 10.90% in AT3. Notably, endothelial cells remained the most abundant type, with no significant change in proportion, consistently representing the predominant cell type in antler tissue.

### 2.3. Subpopulation Analysis of Mesenchymal Cells, Chondrocytes, and Osteoblasts

**Figure 2:**
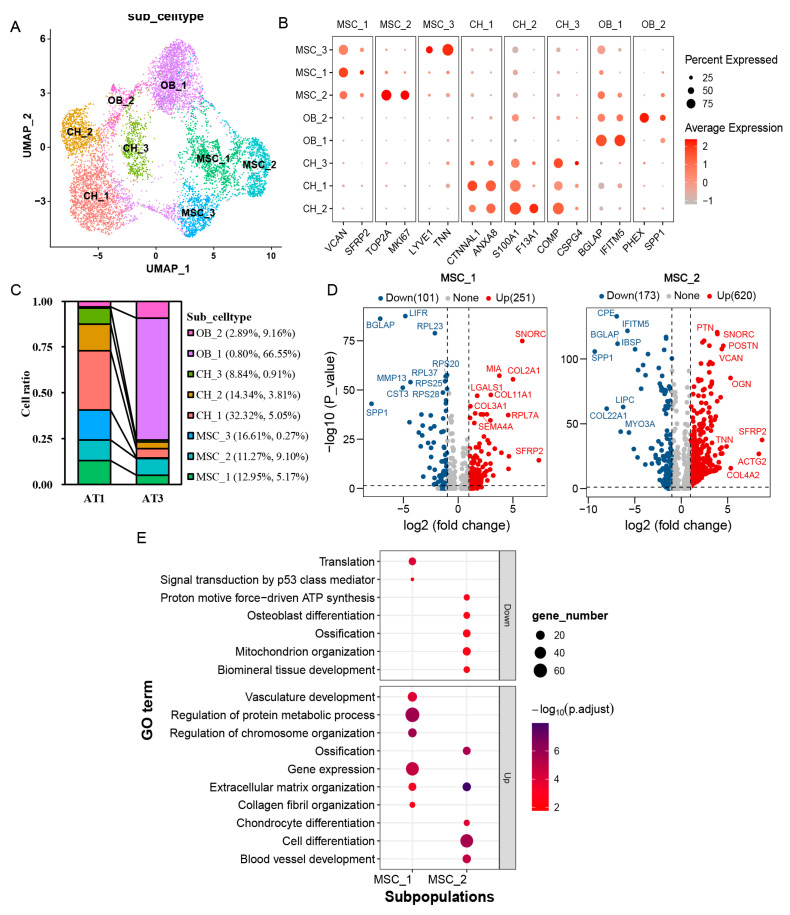
Subpopulation analysis of mesenchymal cells, chondrocytes, and osteoblasts. (**A**): UMAP plot for subpopulations within the mesenchymal cells, chondrocytes, and osteoblasts. Different cell subpopulations labeled by various colors. (**B**): Bubble plot showing marker genes for each cell subpopulation. (**C**): Bar chart showing the proportion of each cell subpopulation in antlers during the growth stage (AT1) and ossification stage (AT3). (**D**): Volcano plot of differential genes between mesenchymal stem cell subpopulations (MSC_1 and MSC_2) in antler tissues during the growth and ossification stages. (**E**): Bubble plot displays the functional enrichment results of differential genes between mesenchymal stem cell subpopulations (MSC_1 and MSC_2) in antler tissues during the growth and ossification stages.

Based on gene expression patterns, mesenchymal cells (MSCs) can be subdivided into three distinct subpopulations, designated as MSC_1, MSC_2, and MSC_3 (Figure 2A). Notably, the MSC_3 subpopulation, characterized by a markedly high expression of *LYVE1* and *TNN*, was predominantly enriched in the AT1 samples (16.61%) (Figure 2B,C). Despite the limited detection of only nine cells (0.27%) in the AT3 sample, these cells retained strong expression of the subpopulation marker *TNN*. In contrast, the number of MSC_2 remained relatively stable, with significantly upregulated proliferation-associated genes (*TOP2A*, *MKI67*). Meanwhile, MSC_1 demonstrated high expression of *VCAN*, a gene related to the extracellular matrix.

We further analyzed the differentially expressed genes (DEGs) between the growth and ossification stages of antlers in the MSC_1 and MSC_2 subpopulations. The results showed that, in the MSC_1 subpopulation, 352 genes were significantly differentially expressed (Figure 2D). Of these, 251 were upregulated, including chondrocyte markers *SNORC*, *COL2A1*, and *MIA*. The remaining 101 were downregulated, including osteoblast markers *BGLAP*, *LIFR*, *SPP1*, *MMP13*, and *CST3*. Functional enrichment analysis revealed that the upregulated genes were primarily involved in the regulation of chromosome organization (*p =* 1.40 × 10^−6^, *DNMT1*, *PARP1*, *CENPF*) and vasculature development (*p =* 9.95 × 10^−5^, *COL8A1*, *NRP1*) (Figure 2E). The downregulated genes were mainly associated with translation (*p =* 7.01 × 10^−5^, *RPL23*, *RPS27L*) and signal transduction by the p53 class mediator (P = 0.0173, *RPL37*, *RPL23*, *RPS20*). In the MSC_2 subpopulation, a total of 793 DEGs were identified. Of these, 620 genes were upregulated, including *SFRP2*, *OGN*, *COL4A2*, *POSTN*, *VCAN*, *TNN*, *COL4A1*, and *PTN*; 173 genes were downregulated, including *SPP1*, *COL22A1*, *CPE*, *BGLAP*, *LIPC*, *IFITM5*, *MYO3A*, and *IBSP*. Functional enrichment analysis revealed that the upregulated genes were primarily associated with cell differentiation (*p =* 2.25 × 10^−6^, *TNN*, *PTN*, *SFRP2*). In contrast, the downregulated genes were related to osteoblast differentiation (*p =* 0.0023, *SPP1*, *BGLAP*, *IBSP*, *SATB2*) and proton-motive force-driven ATP synthesis (*p =* 0.0023, *NDUFA7*, *NDUFB3*, *NDUFA3*, *NDUFB1*).

Chondrocytes were classified into three subpopulations: CH_1, CH_2, and CH_3 (Figure 2A). During the growth stage, the proportions of these subpopulations were 32.32%, 14.34%, and 8.84%, respectively; during the ossification stage, they decreased to 5.05%, 3.81%, and 0.91%. Each subpopulation exhibited distinct functional characteristics: CH_1 expressed genes associated with cell proliferation and adhesion (*CTNNAL1*, *NFE2L3*); CH_2 expressed genes involved in cartilage development (*S100A1*, *SNORC*); and CH_3 expressed extracellular matrix-related genes (*COMP*, *CSPG4*, *COL9A3*, *ACAN*) (Figure 2B).

Unlike mesenchymal cells and chondrocytes, osteoblasts constituted 75.7% of the cell population during the ossification stage and were divided into two subtypes: OB_1 (66.55%) and OB_2 (9.16%) (Figure 2C). OB_1 highly expressed maturation markers *BGLAP* and *IFITM5*, whereas OB_2 expressed genes associated with bone mineralization, including *PHEX* and *SPP1* (Figure 2B).

**Figure 3:**
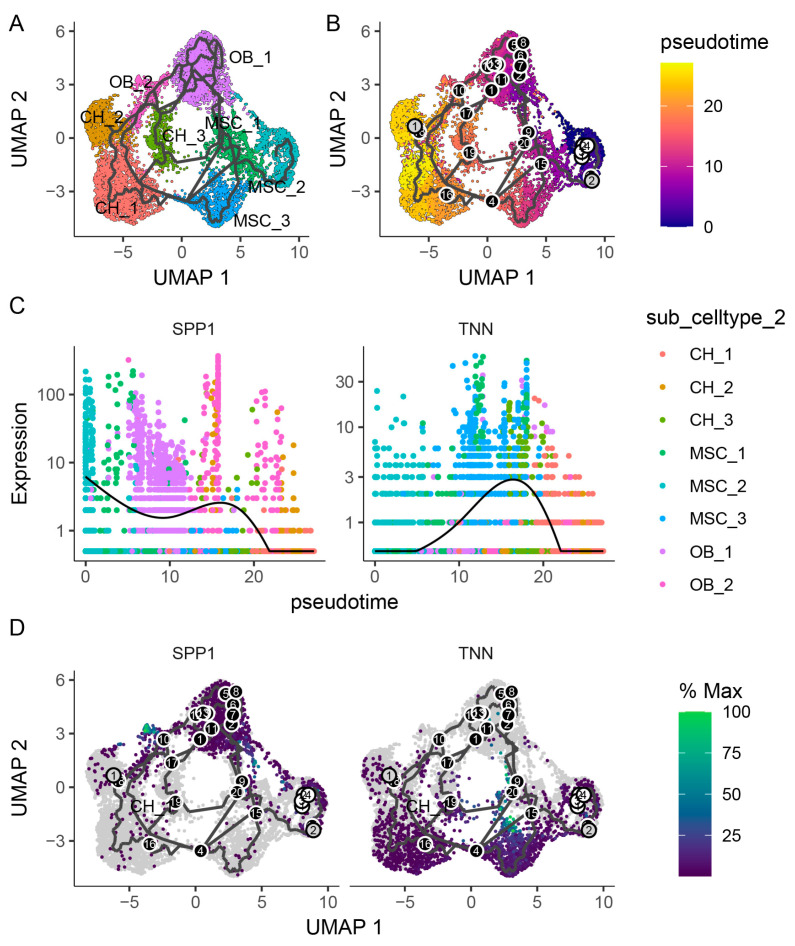
Cell differentiation trajectory analysis of mesenchymal cells, chondrocytes, and osteoblasts. (**A**,**B**): Pseudo-time trajectory plot of mesenchymal cells, chondrocytes, and osteoblasts. (**C**): Expression dynamics of SPP1 and TNN along pseudo-time. (**D**): Feature plot of *SPP1* and *TNN*.

Cell trajectory analysis showed that the mesenchymal cells followed two paths: one leading to osteoblasts and the other to chondrocytes (Figure 3A,B). MSC_2, which highly expressed cell proliferation genes, was likely the initial differentiation point. During the antler growth stage, MSC_2 differentiated into MSC_1, then MSC_3, and, finally, into chondrocytes. However, during the antler ossification stage, the path from MSC_2 to MSC_3 was blocked, preventing chondrocyte differentiation and redirecting cells toward osteoblasts. Pseudo-time analysis revealed that *SPP1* was upregulated in MSC_2 and osteoblasts, while *TNN* was predominantly enriched in the chondrogenic lineage (Figure 3C,D).

### 2.4. Subpopulation Analysis of Endothelial Cells

**Figure 4:**
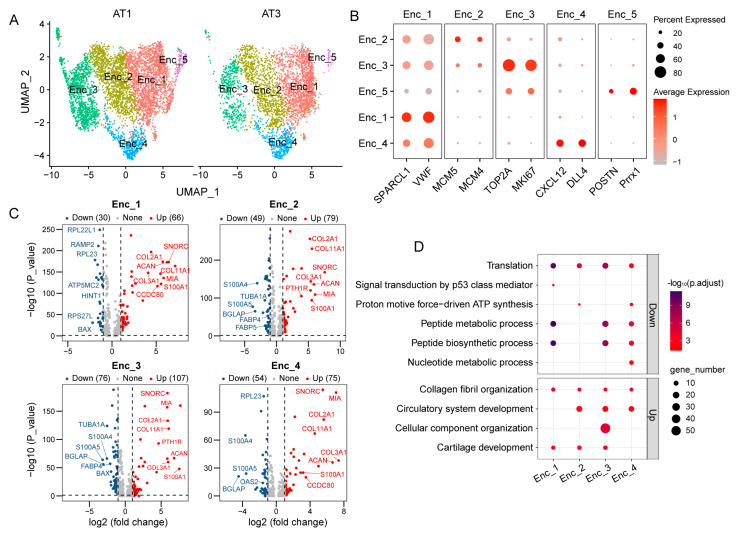
Subpopulation analysis of endothelial cells. (**A**): UMAP visualization of subpopulation analysis of endothelial cells in antler tissues during the growth stage (AT1) and ossification stage (AT3). Different cell subpopulations labeled by various colors. (**B**): Bubble plot illustrating the marker genes for endothelial cell subpopulations (Enc_1, Enc_2, Enc_3, Enc_4, and Enc_5). (**C**): Volcano plot of differential genes between endothelial cells subpopulations (Enc_1, Enc_2, Enc_3, and Enc_4) in antler tissues during the growth and ossification stages. (**D**): Bubble plot illustrating the functional enrichment results of differential genes among endothelial cell subpopulations (Enc_1, Enc_2, Enc_3, and Enc_4) in antler tissues during both the growth and ossification stages.

Endothelial cells constituted a significant proportion of antler tissue, representing 41.14% during the growth stage and 38.63% during the ossification stage (Figure 1E). Based on distinct gene expression profiles, endothelial cells could be categorized into five subpopulations (Enc_1, Enc_2, Enc_3, Enc_4, and Enc_5) (Figure 4A).

Specifically, Enc_1 displayed high expression of *VWF*, a pan-endothelial marker associated with vascular integrity. Enc_2 was enriched with cell cycle regulatory factors *MCM5* and *MCM4*, suggesting their potential role in cell cycle progression. The proliferative nature of Enc_3 was evidenced by the significant upregulation of proliferation marker *MKI67* and DNA topoisomerase *TOP2A*. Enc_4 exhibited arterial endothelial-specific differentiation features, such as elevated *DLL4* expression, indicating its role in arterial remodeling [19]. In contrast, Enc_5 showed higher expression of mesenchymal transition-related molecules *POSTN* and *Prrx1*, potentially involved in endothelial–mesenchymal transition [20] (Figure 4B).

Significantly differentially expressed genes (*p* < 0.05, |log2FC|> 1) in different subgroups of endothelial cells during the growth and ossification stages of antler development were screened. The results revealed that Enc_1 had 66 upregulated and 30 downregulated DEGs; Enc_2 had 79 upregulated and 49 downregulated DEGs; Enc_3 had 107 upregulated and 76 downregulated DEGs; and Enc_4 had 75 upregulated and 54 downregulated DEGs (Figure 4C). Functional enrichment analysis indicated that the upregulated DEGs in all subpopulations were primarily associated with collagen fibril organization. Additionally, the upregulated genes in Enc_2, Enc_3, and Enc_4 were also involved in circulatory system development. The downregulated genes across all subpopulations were mainly related to translation and peptide metabolic processes. Notably, the downregulated genes in Enc_1 were further implicated in signal transduction by the p53 class mediator (Figure 4D).

### 2.5. Combined Analysis of scRNA-seq and Bulk RNA-seq

**Figure 5:**
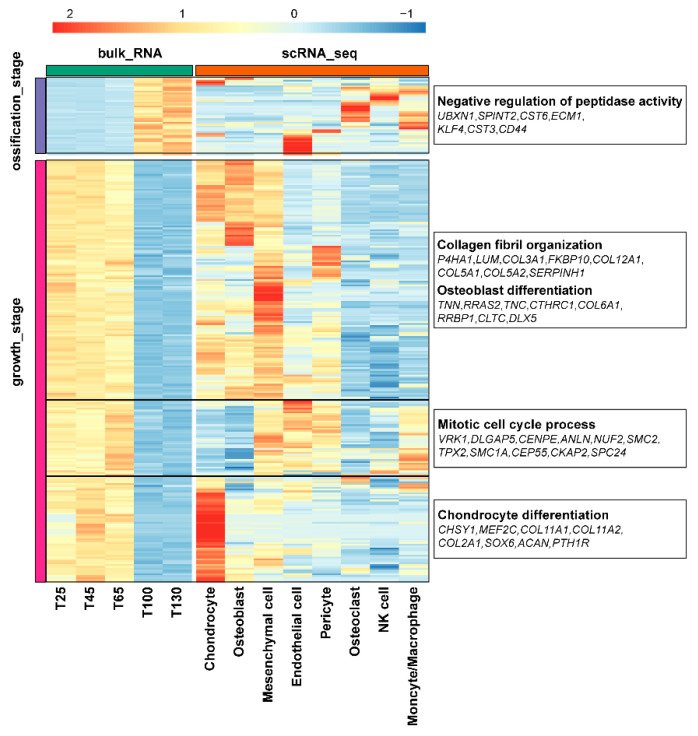
Heatmap of the expression of genes related to the rapid ossification of antlers obtained by integrating bulk_RNA and scRNA-seq analyses. On the right side of the heatmap, the bold text in the boxes represents the functional enrichment results of the corresponding module genes, while the italic text in the boxes represents the names of the corresponding genes.

To validate and extend the differential expression patterns observed in bulk RNA-seq, we integrated the 1717 osteogenesis-associated genes identified from bulk RNA-seq with cell type-specific marker genes derived from our scRNA-seq dataset (Figure 5). Among these, 272 genes were overlapped between the two datasets. Strikingly, 42 of the overlapping genes were upregulated during the ossification stage, including osteoclast-specific genes (*MPP7*, *EMB*, *FRRS1*, *SLC37A2*, *DOCK5*, *RASSF4*, *LGALS3*, *SLC48A1*, *ALDH2*, *SIK1*, and *TIAM1*) and endothelial cell-specific genes (*B3GNT8*, *RNF149*, *FABP4*, *BAZ2B*, *AFDN*, *ECM1*, *CHD7*, *MTM8*, *PALMD*, *DIRF*, and *SYNE2*). Enrichment analysis revealed that these genes were involved in the negative regulation of peptidase activity. The remaining 230 overlapping genes exhibited stage- and cell type-specific expression patterns. Some genes were highly expressed in mesenchymal cells, chondrocytes, and osteoblasts, related to collagen fibril organization and osteoblast differentiation. Another subset was highly expressed in mesenchymal cells, endothelial cells, and pericytes, primarily involved in the mitotic cell cycle process. Additionally, several chondrocyte marker genes were identified, which were involved in chondrocyte differentiation.

## 3. Discussion

To elucidate the molecular regulatory mechanisms underlying rapid bone formation in antlers, we previously conducted comprehensive analyses using proteomic and transcriptomic techniques. These efforts led to the preliminary identification of key regulatory factors involved in this process. However, the existing data are not sufficient to fully clarify the underlying regulatory mechanisms. Given the recent advancements in single-cell omics technologies, we aim to further investigate the rapid bone formation mechanism in antlers from a single-cell perspective.

In this study, we employed single-cell transcriptomic technology and identified eight distinct cell types in antler tissue during the growth and ossification stages. These cell types included not only well-characterized antler mesenchymal stem cells but also chondrocytes, osteoblasts, a substantial number of endothelial cells, and minor populations of osteoclasts, monocytes/macrophages, and NK cells. As anticipated, significant differences were observed between the growth and ossification stages. During the growth stage, mesenchymal cells and chondrocytes predominated, facilitating rapid antler growth. In contrast, during the ossification stage, the number of mesenchymal cells and chondrocytes sharply declined, while osteoblasts became the dominant cell type. Notably, the proportion of osteoclasts increased from 1.64% during the growth stage to 10.9% during the ossification stage. Osteoclasts are the sole cell type capable of degrading bone and play a critical role in bone metabolism. The marker gene MPP7 was found to exhibit constitutive expression in human bone-derived cells during osteogenesis, and its knockout has been shown to lead to reduced bone mass [21,22].

The rapid ossification process in antlers occurs in a manner analogous to endochondral ossification. Specifically, mesenchymal cells continuously proliferate and differentiate into chondrocytes, which then secrete chondroid matrix to form a cartilage layer. This layer is subsequently gradually replaced by bone. During the growth stage of antlers, the primary cells are mesenchymal cells and chondrocytes, with a relatively low number of osteoblasts. This leads to the speculation that the events occurring during the growth stage of antlers correspond to the initial stage of endochondral ossification, namely, the formation of the cartilage layer. An increase in the number of osteoblasts and osteoclasts indicates the progression to the stage during which cartilage is gradually replaced by bone.

Mesenchymal stromal cells (MSCs) are the key cellular component of bone regeneration since they can differentiate into chondrogenic and osteoblastic lineages. Using trajectory analysis, we uncovered a dynamic transition in MSC fate commitment between two distinct developmental phases: the chondrogenesis-dominated antler growth stage and the osteogenesis-dominated ossification stage. MSC_2 cells exhibited high expression of cell proliferation-related genes (*TOP2A*, *MKI67*, *CENPF*, and *UBE2C*). Furthermore, the population of these cells remained relatively stable between the growth stage and the ossification stage, leading us to designate them as the starting point of the cellular differentiation trajectory. During the growth stage, MSC_2 cells showed the upregulation of genes (*POSTN*, *VCAN*, *TNN*, *PTN*) critical for MSC self-renewal and differentiation during bone healing [23,24,25,26]. Furthermore, *TNN* served as a marker gene for the MSC_3 subpopulation, which was uniquely present during antler growth. Studies have demonstrated that *TNN* can suppresses osteoblast proliferation and differentiation during endochondral ossification. Furthermore, during the process of endochondral ossification, *TNN* has the ability to suppress osteoblast proliferation and differentiation that are mediated by the canonical WNT signaling pathway [27], suggesting that its upregulation may drive chondrogenesis by inhibiting osteogenesis. During the process of osteoblast differentiation, there was an upregulation in the expression of genes such as *SPP1*, *IBSP*, *BGLAP*, and *COL1A1*. Among these, *SPP1* and *IBSP* were primarily expressed in immature osteoblasts, while matured osteoblasts strongly expressed *BGLAP* and *COL1A1* [28]. Therefore, the upregulation of osteoblast differentiation-related genes led to the differentiation of the MSC_2 cell subpopulation into osteoblasts.

The differences in gene expression between the MSC_1 cell subpopulation during the growth and ossification stages were similar to those found in MSC_2. Specifically, during the growth stage, MSC_1 expressed more chondrocyte markers such as *SNORC* [29,30], *COL2A1*, and *MIA*. Among these, *MIA* influences the role of *BMP-2* and *TGF-β3* during mesenchymal stem cell differentiation, supporting the formation of the chondrocyte phenotype while inhibiting osteogenic differentiation [31,32]. During the ossification stage, in addition to expressing higher levels of osteogenic markers, genes involved in signal transduction by the p53 class mediator, such as *RPL37*, *RPL23*, *RPS20*, and *RPL26*, were also identified. These genes can regulate the stability and function of p53 by binding to *MDM2*, thereby influencing processes such as cell cycle progression, apoptosis, metabolism, and tumor suppression [33,34]. Therefore, we hypothesized that the high expression of these genes directly contributed to the sharp decline in the MSC_1 cell population during the ossification stage.

The second major phase of endochondral ossification involves the replacement of cartilage by bone, during which, in addition to osteoblasts, another type of cell becomes involved: osteoclasts. These cells highly express genes such as *ACP5*, *MMP9*, *ATP6V0D2*, and *ATP6V1B2*. Among them, *MMP9* can degrade type IV and V collagens, while *ATP6V0D2* and *ATP6V1B2* maintain the acidic environment necessary for osteoclasts to perform bone resorption [35,36]. This suggests that, during the ossification stage of antler development, osteoclasts are responsible for degrading the calcified cartilage matrix, preparing the way for the accumulation of osteoblasts and bone cells. Furthermore, among the genes upregulated during the ossification stage, *MPP7*, *EMB*, *FRRS1*, *SLC37A2*, *DOCK5*, *RASSF4*, *LGALS3*, *SLC48A1*, *ALDH2*, *SIK1*, and *TIAM1* are primarily expressed in osteoclasts. Notably, *DOCK5* gene expression can be activated by RANKL, regulating bone resorption [37,38], while *SIK1* is capable of modulating osteoclast differentiation [39]. These findings further underscore the significance of osteoclasts in the rapid ossification process of antlers.

Indeed, it has been demonstrated that many bone formation processes, including fracture healing, distraction osteogenesis, and skeletal development, heavily rely on angiogenesis [40]. Naturally, the rapid ossification of antlers is also closely related to angiogenesis [41]. In this study, we identified endothelial cells that highly express *PECAM1* and *VWF*. During blood vessel development and neovascularization, *PECAM1* participates in adhesion and signal transduction between endothelial cells [42]. Moreover, the marker genes of these cells are enriched in pathways involved in blood vessel development, suggesting that these cells are likely vascular endothelial cells. Unlike chondrocytes and osteoblasts, endothelial cells are an essential component of antlers both during the growth and ossification stages. However, there is a difference: chondrogenesis-related genes are upregulated in endothelial cells during the growth stage, while genes related to signal transduction by the p53 class mediator are upregulated during the ossification stage, such as *BAX*, *HINT1*, *RPS27L*, and *RPL26*. These genes can regulate the stability and function of p53, thereby influencing processes such as cell cycle progression, apoptosis, metabolism, and tumor suppression. Therefore, during the ossification stage, the cell cycle of these endothelial cells is inhibited, further affecting their proliferation. Although our research has made some progress, there are still certain limitations. In this study, the samples from the growth stage and the ossification stage were all obtained from the same experimental animal individual, and no biological replicates were set up. This might have affected the reliability and generalizability of the research results.

## 4. Materials and Methods

### 4.1. Sample Selection and Preparation

A 4-year-old male sika deer was selected from the deer farm of Institute of Special Animal and Plant Sciences, Chinese Academy of Agricultural Sciences. The deer was first anesthetized with 5% xylazine at 0.5 mg/kg body weight intramuscularly. Antlers were harvested on the 60th day (antler growth stages, AT1) and the 90th day (antler ossification stage, AT3) following the shedding of the previous hard antlers. The antlers were washed with PBS buffer to remove contaminants, and longitudinal slices of approximately 2 mm thickness were uniformly obtained from the apical 3 cm region of the antler tips using a sterile surgical scalpel under aseptic conditions. All animal experimental protocols were approved and authorized by the Chinese Academy of Agricultural Sciences Animal Care and Use Committee (NO. ISAPSAEC-2023-007).

### 4.2. Cell Preparation

The samples were dissociated by gentle agitation in a 37 °C digestion enzyme solution (2 mg/mL trypsin and 1 mg/mL collagenase IV) for 30 min. The digestion was terminated by washing three times with ice-cold blocking solution (10% FBS in PBS without Ca^2+^/Mg^2+^). The digested samples were filtered through 40 µm strainers to remove impurities, followed by centrifugation at 300× *g* for 5 min. The cell pellet was re-suspended in 100 μL of PBS solution containing 0.04% BSA. The single-cell suspension was then assessed using Trypan Blue staining and fluorescent cell counting to ensure that the viability exceeded 80%. To achieve the target of 10,000 cells per sample, cell concentration was adjusted to 1000 cells/μL, and 10 μL of suspension was loaded into the Chromium Controller (10× Genomics).

### 4.3. Single-Cell RNA Sequencing

Single-cell suspensions were processed for droplet-based scRNA-seq, wherein individual cells were encapsulated into emulsion droplets utilizing the Chromium Controller (10× Genomics). The preparation of scRNA-seq libraries was carried out with the Chromium Single Cell 3′ Reagent Kit (10× Genomics, PN-10000075), following the protocol provided by the manufacturer. Library quality and quantity were assessed using the Qubit 2.0 Fluorimeter (Life Technologies, Carlsbad, CA, USA) and the Bioanalyzer 2100 (Agilent Technologies, Santa Clara, CA, USA). Sequencing was performed on the Illumina HiSeq PE150 platform (Illumina, San Diego, CA, USA) with paired-end 150 bp reads, targeting a total sequencing depth of 300 Gb per sample.

### 4.4. Data Analysis

The raw FASTQ data obtained from single-cell RNA sequencing were aligned to the sika deer reference genome [43] using the Cell Ranger software (10× Genomics). Subsequently, Cell Ranger performed filtering, barcode counting, and UMI counting to generate a cell–gene expression matrix. The Seurat software (version 4.3.0) was employed for cell re-filtering based on gene detection numbers, dimensionality reduction, and clustering, as well as the identification of differentially expressed genes. Cells expressing fewer than 200 or more than 2500 genes, as well as those with over 10% mitochondrial gene expression, were excluded from the analysis. The “IntegrateData” function in Seurat was utilized to integrate datasets and mitigate batch effects. The “NormalizeData” function in Seurat was employed to normalize the data using the LogNormalize method with a scaling factor of 10,000. Subsequently, the “FindVariableFeatures” function was utilized to identify 2000 highly variable genes. The “ScaleData” function was used to normalize gene expression levels, preventing biases in cell clustering due to extreme gene expression. The “RunPCA” function performed the initial dimensionality reduction, the “JackStraw” function calculated *p*-values for genes in each principal component, and the “FindNeighbors” and “FindClusters” functions conducted a clustering analysis. The “RunUMAP” function performed nonlinear dimensionality reduction and generated a UMAP clustering plot.

### 4.5. Cell Annotation and Differential Gene Expression Analysis

The “FindAllMarkers” function in Seurat was used to identify DEGs with the following parameters: log2 fold-change threshold > 0.25, adjusted *p*-value < 0.05, and minimum percentage of cells expressing the gene in either group set to 10%. The Wilcoxon rank-sum test was employed as the statistical method for DEG detection. Cell types were annotated using single-cell profiles from the CellMarker2.0 database and the relevant literature. GO and KEGG enrichment analyses for DEGs were conducted using the clusterProfiler package (version 4.2.0) in R. Cell trajectory analysis was performed using the Monocle3 package (version 1.3.1).

## 5. Conclusions

In summary, this study revealed phase-specific regulatory programs driving antler morphogenesis. During the growth stage, genes related to vasculature, collagen organization, and cell differentiation were upregulated. During the ossification stage, genes involved in osteoblast differentiation, p53 signaling, and ATP synthesis were activated. Notably, osteoclast- and endothelial cell-specific genes were upregulated during the ossification stage, while the growth stage’s genes were linked to collagen organization and chondrocyte differentiation. Overall, our findings offer valuable insights into the molecular mechanisms driving rapid antler osteogenesis.

## Data Availability

The data presented in this study are available upon request from the corresponding author.

## Supplementary Materials

The following supporting information can be downloaded at https://www.mdpi.com/article/10.3390/ijms26062642/s1.
